# An IoT-Based Breeding Egg Identification and Coding System for Selection of High-Quality Breeding Geese

**DOI:** 10.3390/ani12121545

**Published:** 2022-06-14

**Authors:** Yanjun Zhang, Yujie Ge, Tian Yang, Yangyang Guo, Jian Yang, Jiawen Han, Daoqing Gong, Hong Miao

**Affiliations:** 1 College of Mechanical Engineering, Yangzhou University, Yangzhou 225127, China; geyj.1127@gmail.com (Y.G.); jsntrdmtyt@163.com (T.Y.); guoyangyang_gyy@163.com (Y.G.); jianyang@yzu.edu.cn (J.Y.); hjw18852724546@163.com (J.H.); mh0514@163.com (H.M.); 2 College of Animal Science and Technology, Yangzhou University, Yangzhou 225127, China; dqgong@yzu.edu.cn

**Keywords:** selection of high-quality breeding geese, IoT, RFID, improved SSD, information correspondence

## Abstract

**Simple Summary:**

In the process of breeding geese, manually recording data causes the problems of missing and confusing characterization data; furthermore, manual intervention can lead to the stress response of breeding geese and affect the laying efficiency of breeding geese. In this study, we tried to combine the Internet of things and computer image technology to improve the accuracy of data recording, so as to achieve an accurate correspondence between breeding goose individual data and egg-laying data. Therefore, we developed and tested a breeding egg identification and coding system to realize the selection of high-quality breeding geese. The test results showed that the system realized a correspondence of 97.8% between breeding goose individual data and egg-laying data. The system realized the information recording of breeding geese under nonmanual intervention, realized the correspondence between individual data and egg-laying data, and improved the health and welfare of breeding geese.

**Abstract:**

The selection of breeding geese requires the recording of egg production information to correspond to the identity of the breeding geese. However, due to the special physiological characteristics of breeding geese, manual recording in practice can affect the egg-laying performance of breeding geese and can also lead to problems of missing and confusing individual breeding goose data with the number of eggs laid by the geese. For contactless recording of breeding goose identity and egg production information for high-quality breeding, this paper proposes an Internet of things (IoT)-based breeding egg identification and coding method for the selection of high-quality breeding geese. At the sensing level, we deployed a radiofrequency identification (RFID)-based sensor. Each breeding goose wore a foot ring RFID tag on its leg, and the individual information was read by foot ring RFID readers placed at the bottom of the devices. Individual information was uploaded to the cloud server for database management through structured query language (MySQL). The target detection modules were mounted on top of the devices, and the breeding geese and eggs were detected in the delivery rooms by an improved single-shot multi-box detector (SSD) target detection algorithm. The egg body limit transmission device and contactless coding device were activated only in the case of breeding eggs, and the breeding goose information was printed on the egg bodies in the form of quick response codes (QR codes), which enabled the breeding egg information to correspond with the breeding goose information. An evaluative experiment was performed using a system for the selection of high-quality breeding geese, with web cameras and a cloud monitoring platform. The breeding geese were allowed 14 days to become accustomed to the experimental environment before monitoring began. The evaluative experiment results showed that the pass rate of egg body coding reached 98.25%, the improved SSD algorithm was 8.65% more accurate and 62.6 ms faster than traditional SSD, and the accuracy rate corresponding to the individual information of the breeding geese and the surface information of the goose eggs was 97.8%. The experimental results met the requirements of accurate marking of individual information of breeding geese, which can provide technical support for the selection of high-quality breeding geese.

## 1. Introduction

Goose breeding has a long history in China [1]. Unlike other poultries, breeding geese have a stress response. The breeding method is based on manual selection and breeding, which is labor-intensive for the staff, and the manual recording of individual data for breeding geese is prone to problems such as omission and confusion regarding the number of eggs laid and the characterization data [2,3]. This particular habit also limits the selection of breeding geese to free range or flat rearing, making it more difficult to collect egg production data [4,5]. In order to advance the process of intelligent goose breeding, contactless data collection on breeding geese becomes a key prerequisite which allows for the selection of high-quality breeding geese. Therefore, it is especially important to propose an efficient and accurate contactless data collection device for breeding geese.

With the introduction of RFID technology to study the individual identification and traceability of animals, as proposed by Luis et al. [6], RFID has become one of the most popular technologies for identifying individual animals and has been used in farming research on animals such as pigs, chickens, cattle, and sheep [7,8,9,10,11,12,13]. Siegford et al. [14] suggested RFID and radio signal strength (RSSI) technology to achieve individual identification in large-scale poultry farming processes. Egg collection is an important link in egg production monitoring and breeding. Burel et al. [15] and Icken et al. [16] designed egg-laying crates with funnels to collect eggs, increasing buffering and reducing egg bruising; they used RFID identification systems to achieve a unique correspondence between data and individual chicken tags, accurately collecting individual egg-laying data from chickens. However, the presence of chickens entering the laying box without laying eggs makes the egg-laying monitoring data inaccurate, which leads to incorrect chicken–egg pairing. Chien et al. [17] also designed a smart egg-laying box for hens on this basis, with a special sloping surface located at the bottom of the box allowing eggs to roll through an inclined tray into an egg collection tube at the back of the box. A pressure measuring element was installed under the egg collection tube as an egg detection sensor and an egg weight sensor, whereby RFID identification technology and photoelectric sensing technology were combined to determine whether the hens have left the laying room; however, collection by tilting and rolling inevitably caused eggs to break. At the same time, the device could only count the number of eggs laid, and the correspondence between poultry individual information and egg-laying information could not be realized.

The disadvantage of RFID for quality selection is the inability to work on traceability. With the development of IoT, there have been several applications of IoT in agriculture, such as agricultural environmental monitoring, livestock breeding, and poultry farming [18,19]. In the case of the poultry farming industry, the IoT has enabled the effective collection of egg production numbers and characterization data from caged poultry such as chickens and ducks. Voulodimos et al. [20] used RFID technology to achieve livestock tracking and management by establishing a data repository through which, in addition to RFID tag numbers associated with animal data records, the use of rewritable tags was introduced for storing information to be used to identify animals in case of loss. Srbinovska et al. [21] designed a greenhouse vegetable monitoring system, based on wireless sensor network technology, to enable the monitoring of environmental parameters such as temperature, humidity, and carbon dioxide concentration in vegetable greenhouses. Miso et al. [22] proposed a combination of cloud computing and a wireless sensor network for environmental monitoring by transmitting sensor data to the cloud for cloud computing through the wireless sensor network; the experiment proved the operability of the wireless sensor network. Rodrigo et al. [23] developed an integrated hardware and software automation system for continuous seed monitoring and recording operations through ColoT as an architecture developed for sensory processing of devices in the IoT to monitor and record data throughout the testing process. The IoT technologies mentioned above were only used in agricultural monitoring and were not used for traceability work to enable the correspondence between egg production data and characterization data.

In this paper, we designed an IoT-based breeding egg identification and coding system for the selection of high-quality breeding geese combined with RFID, target detection, and IoT technologies. The combination of the target detection module and RFID in the device helped us to determine the identity of the breeding geese entering the delivery rooms and the situation inside the rooms, as well as transmit the breeding goose identity information to the cloud service. In the event that only eggs were present in the delivery rooms, the egg limit transfer device and the contactless coding device were activated, and the information stored in the cloud server was sprayed on the eggs in the form of QR codes. Our proposed system enabled breeders of geese to use the egg yield of individual breeding geese as a means of determining which breeding geese should be culled. This was used for the purpose of selecting high-quality breeding geese.

## 2. Materials and Methods

**Figure 1:**
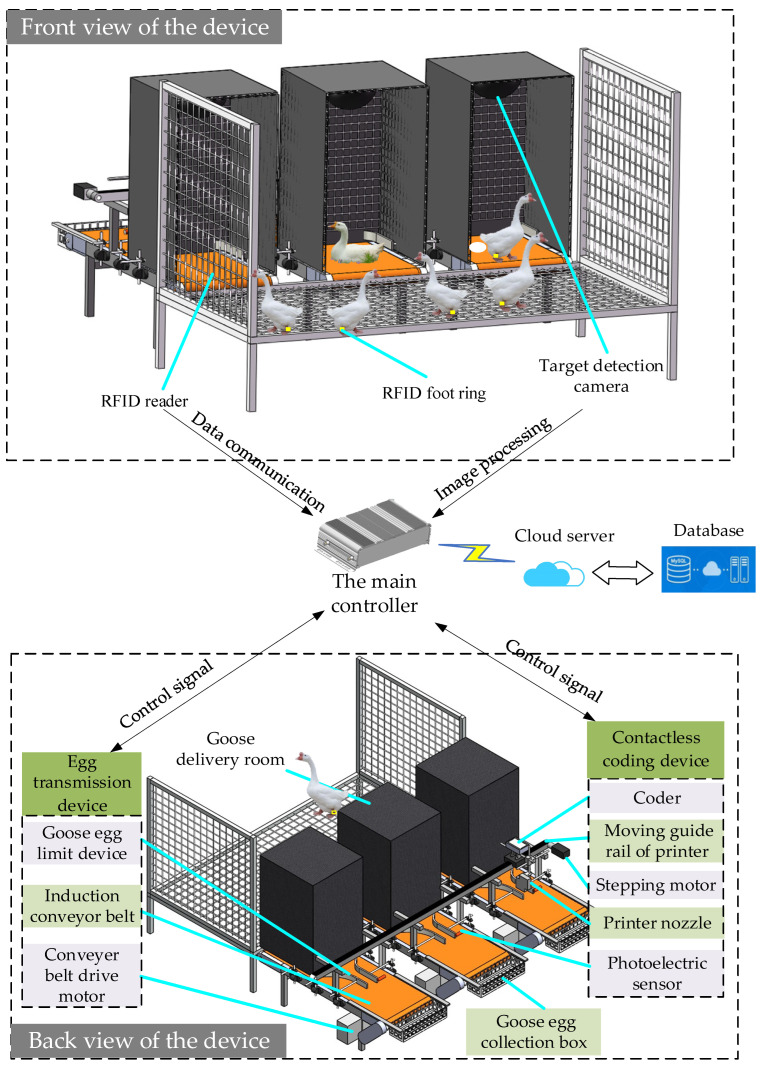
Operation mechanism of breeding egg identification and coding system.

As shown in Figure 1, the overall device was an open structure, with a length of 500 mm, a height of 800 mm, and a width of 470 mm, allowing geese to enter and leave the delivery room freely. By adjusting the delivery room space, it was ensured that only one goose could lay eggs comfortably, avoiding the identification error caused by multiple geese occupying the nest. The RFID reader under the turf read the RFID signal on the foot rings of the breeding geese to determine the identity of the breeding geese and the time when the breeding goose entered and left the delivery room. The main controller stored the data in the established MySQL database through communication with the cloud to store the identity information of the breeding geese. A gap of 75 mm was left at the bottom of the delivery room to transfer goose eggs from the delivery room area to the coding area. At the same time, in order to meet the habit of geese laying eggs in quiet, dark, and dry places [24,25,26,27], artificial turf was sewn on the surface of the induction conveyor belt and the delivery room was wrapped with black cloth to create a real laying environment for breeding geese and improve the comfort and laying efficiency of breeding geese. When the target detection module detected that there were only goose eggs in the delivery rooms, the egg body limit transmission devices and contactless coding devices were started, and the breeding geese identity information was printed on the corresponding egg bodies in the form of QR codes to realize one-to-one correspondence between the laying information and the breeding geese identity information.

**Figure 2:**
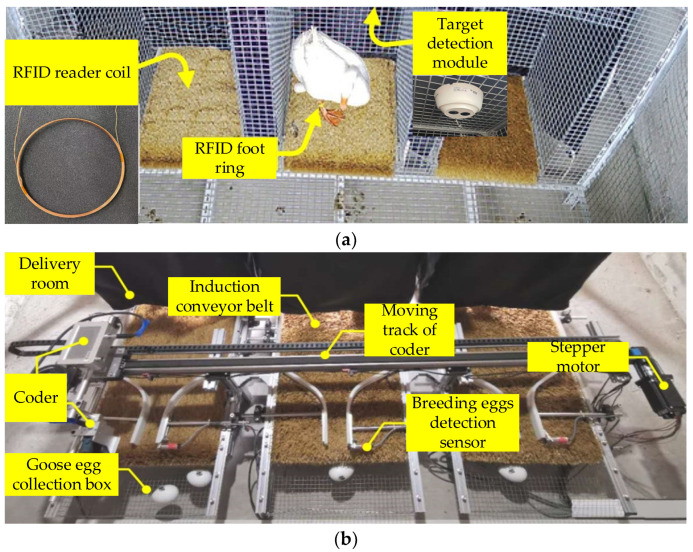
The prototype of goose breeding egg identification and coding system. (**a**) Front view of the system. (**b**) Back view of the system.

We constructed the breeding egg identification and coding system for high-quality breeding geese, and we conducted experiments in the goose breeding base of Yangzhou University, Jiangsu, China. The main controller had a Celeron J1800 CPU, graphics620 core graphics card, and 8 GB of memory. The prototype of the goose breeding egg identification and coding system is shown in Figure 2.

### 2.1. RFID Module

The RFID module was used in the whole system to identify the breeding goose and upload the identity information to the cloud. The RFID module consisted of an RFID reader (operating frequency: 134.2 MHz; label format: FDX-B; standard: ISO11784/85; reading time: <100 ms; read distance: >25 cm) and RFID foot rings which were worn by breeding geese. The RFID readers obtained the RFID tag values, uploaded the tag information to the cloud, and identified and recorded the individual identity information of the breeding goose. The system combined the time sequence before and after the RFID tag value was read by the card reader to judge whether the breeding goose entered the delivery room or the delivery room. When the breeding geese entered the breeding room to lay eggs, the RFID tags worn on the breeding geese’s feet entered the antenna magnetic field of the readers. The readers transmitted energy to the RFID tags through inductive coupling. After the RFID tags obtained the energy, the breeding goose identity information in the tag was transmitted to the readers, which decoded and uploaded it to the cloud to obtain the breeding goose identity information.

### 2.2. Target Detection Module

**Figure 3:**
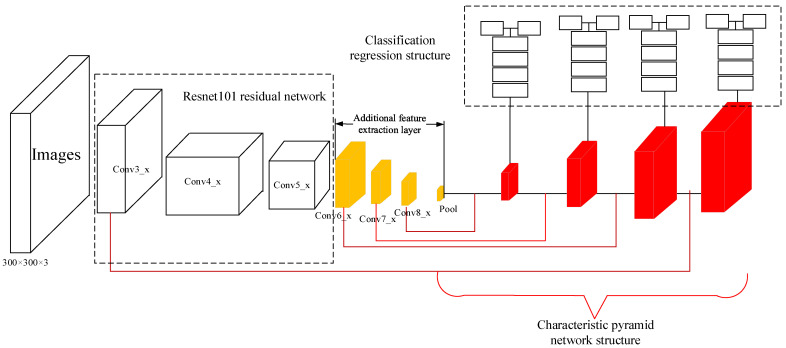
Improved SSD structure.

In the breeding process, in order to avoid the stress response of breeding geese caused by the egg body conveyors, it was very necessary to detect the targets in the delivery rooms before the operation of the conveyor. Current mainstream target detection algorithms are mainly divided into two categories. The first category is based on the candidate region. Firstly, the recognition region is generated by the region of interest (ROI). Then, the convolutional neural network is used for feature extraction and classification. Finally, the size and position of the recognition region frame are adjusted by linear regression. Region-based convolutional neural network (R-CNN) [28] and fast region-based convolutional neural network (fast R-CNN) [29] are representative algorithms. The second category is based on regression. This algorithm directly regresses the position of the object to be tested from the image and completes the classification with a fast recognition speed. Representative algorithms are You Only Look Once (YOLO) [30,31] and SSD [32]. Since this research was aimed at detecting a dynamic video, and since the goose egg distribution position in the video was not fixed, thus influencing the setting of ROI areas, a target detection algorithm based on the candidate region was not suitable for this study. Compared with YOLO, SSD combines not only the regression idea in YOLO, but also the anchor mechanism in fast R-CNN, which takes into account both speed and accuracy. Therefore, this paper improved the SSD to meet the target detection requirements of this study. The specific structure is shown in Figure 3.

**Figure 4:**
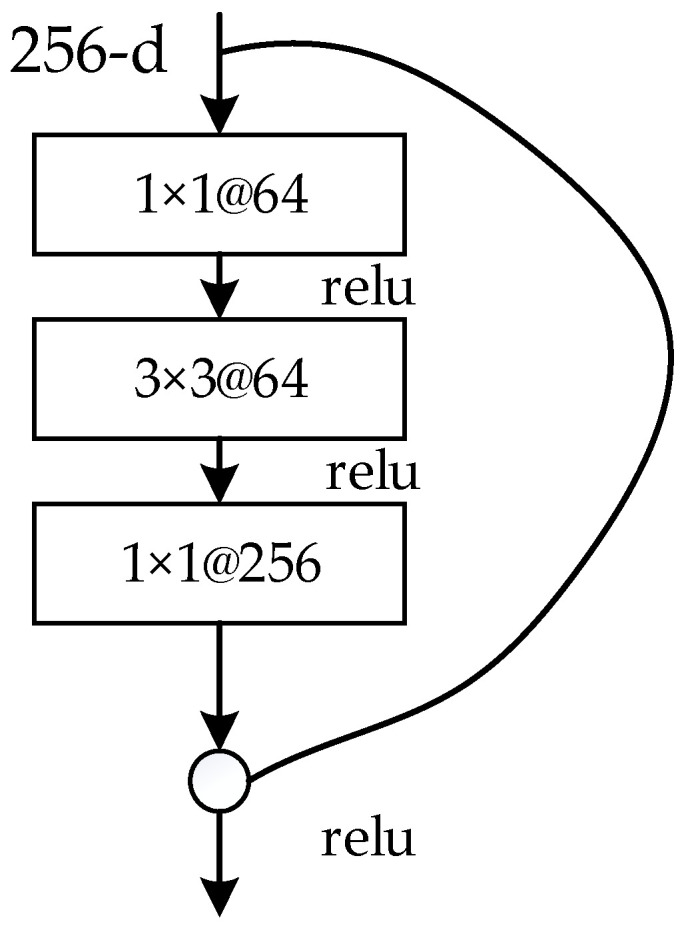
Resnet101 residual network connection mode.

As shown in Figure 3, we used the ResNet101 residual network to replace the original VGG-16 basic network. In order to improve the operation accuracy and reduce the complexity of the model, this paper adopted the cross-layer connection method, as shown in Figure 4. The specific number of layers was calculated as conv_2x, conv_3, conv_4, and conv_5. The residual units were added and multiplied by three layers of connection, plus two full connection layers to realize classification and regression.

**Figure 5:**
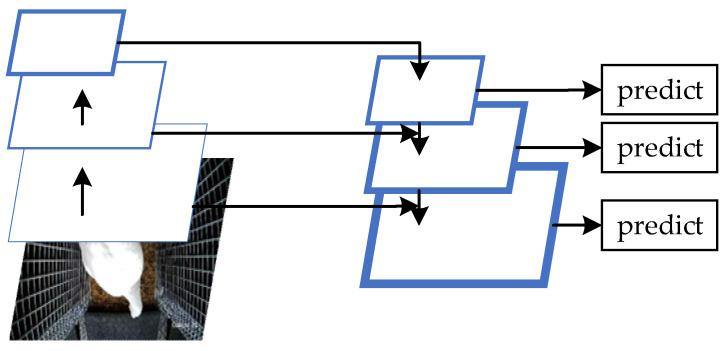
Characteristic pyramid network diagram.

**Figure 6:**
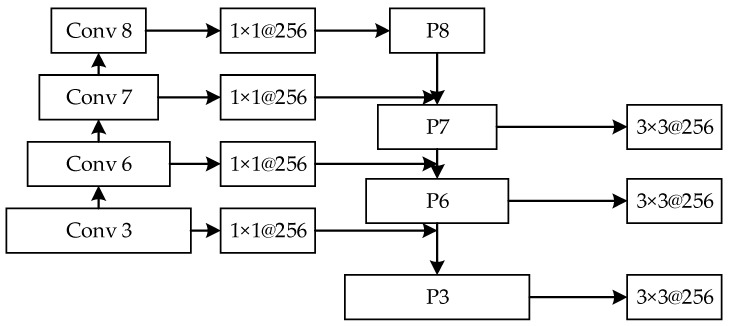
Characteristic pyramid network connection mode diagram.

At the same time, in order to realize the fusion of high-level image features and low-level image features, the feature pyramid could realize prediction on any feature layer of the image. The network structure of the feature pyramid is shown in Figure 5. The blue box represents the feature map, and the thickness of the box represents the semantic strength. A thicker box denotes stronger semantics. The feature pyramid network first performs a bottom-up feature convolution operation on the image, and then performs a top-down up sampling process to enrich the feature information of the low-level image, making predictions for each feature layer. As this study aimed to detect two different scales of target objects, breeding geese and goose eggs, as shown in Figure 6, the network structure was built after the additional feature extraction layer according to the feature pyramid network, which was divided into four parts: bottom-up, top-down, horizontal connection, and prediction output. Firstly, the bottom-up part was the feedforward calculation of the convolution network, which calculated the feature level composed of different proportions of mappings. The convolution layers used in this study were conv3_x, conv6, conv7, and conv8, and the feature output was {C3, C6, C7, C8} after bottom-up convolution. Secondly, the top-down part was an up-sampling process, which transferred the stronger semantic features of breeding geese and goose eggs in the high-level image to the low-level high-resolution feature images, and then horizontally connected the bottom-up process to enhance the feature information of the high-level images. The third part was the horizontal connection between the top-down process feature image and the bottom-up feature image through a 1 × 1 convolution kernel to reduce the number of feature images. In the fourth part, at the beginning of the iteration, a 1 × 1 convolution kernel needed to be added to C8 to generate a low-resolution image P8. In order to reduce the aliasing effect caused by up-sampling, the combined feature images of each layer needed to pass through the convolution kernel to generate the final prediction feature output {P3, P6, P7, P8}.

In order to evaluate the constructed model, this paper used the accuracy rate (*P_i_*), recall rate (*R_n_*), average precision (*AP*), and mean average precision (*MAP*) as evaluation indicators, and their specific expressions are presented in Equations (1)–(4).

(1) $$P_{i}=\frac{R_{s_{i}}}{R_{s_{i}}+F_{s_{i}}},$$

(2) $$R_{n}=\frac{R_{s_{n}}}{R_{s_{n}}+F_{N_{n}}},$$

where *R_Si_* is the case of a correctly detected breeding goose and goose egg, *F_Si_* is the case of no breeding goose and goose egg being detected, *S* is the number of categories, and *F_Nn_* is the number of samples incorrectly divided into negative samples.

(3) $$AP=\frac{1}{T}\;\sum_{K=i}^{N}\;P\left(k\right)\;R\left(k\right),$$

where *T* is the total number of images in the dataset, and *K* is the threshold number.

(4) $$MAP=\frac{\sum_{i=1}^{H}\;AP}{S}.$$

### 2.3. Transmission Coding Module

When the target detection module detected that there was only a breeding egg in the delivery room, it started the egg body limit transmission device to transmit the breeding egg to the coding place. The contactless coding device printed the breeding goose information on the egg surface in the form of a QR code without contacting the egg surface. The goose breeders got the matching breeding goose information by scanning the QR codes on the eggs, so as to realize the breeding of high-quality breeding geese. In the process of goose egg production, goose eggs can be displayed in the delivery rooms at different attitudes, which has a certain randomness. In order to realize the integrity of goose egg surface coding, it was necessary to ensure that the long axis direction of the egg body was basically parallel to the conveying direction of the induction conveyor belt. Therefore, in the process of goose egg transmission, it was necessary to limit and adjust the attitude of goose eggs, so as to achieve the best inkjet effect on the goose egg surface. The egg body limit transmission device was symmetrically installed above the induction conveyor belt to avoid damage to the turf caused by contact with the induction conveyor belt. The criterion of correct attitude adjustment was the deviation between the long axis direction of goose egg body and the conveying direction of the induction conveyor belt, which was set as ±5° in this study. The total length of the limit device, the width of the limit pipe, and the deflection angle of the goose egg can affect the accuracy of the final posture adjustment of the goose egg. Therefore, a quadratic polynomial regression model was established for the success rate of goose egg attitude adjustment to determine the specific size of the limit conveyor. The specific equation is shown in Equation (5).

(5) $$\eta =98-0.75A+1.25B+C+AB-0.5AC-0.5BC-3B^{2}+0.5C^{2},$$

where *A* is the deflection angle of the goose egg, *B* is the width of the limit pipe, *C* is the total length of the limit device, and η is the success rate of goose egg attitude adjustment.

The breeding eggs arrived at the contactless coding device through the egg limit transmission device, and the photoelectric sensor transmitted the electrical signal to the main controller. The main controllers used the RS485 serial port communication to transmit the label information and egg-laying date of the breeding goose to the coder. The coders compiled the information and applied it via inkjet onto the surfaces of the goose eggs in the form of QR codes. The QR codes were marked on the surfaces of goose eggs through the nozzle to provide data support for identifying and screening high-quality and high-yield goose individuals. The mass of the printer nozzle was 2.4 kg, and the weight of the printer was 2.1 kg. Due to the low overall load, a stepper motor was selected for driving. In this study, a two-phase DC 42 mm stepper motor and PMC006B4 small stepper motor driver were selected. The movement of the printer sent pulse signals to the motor driver through the main controllers, and the guide rail was driven to move through the rotation of the motor, so as to realize the lateral movement of the printer.

## 3. Results and Discussion

### 3.1. Characterization Test of Transmission Coding Module

**Figure 7:**
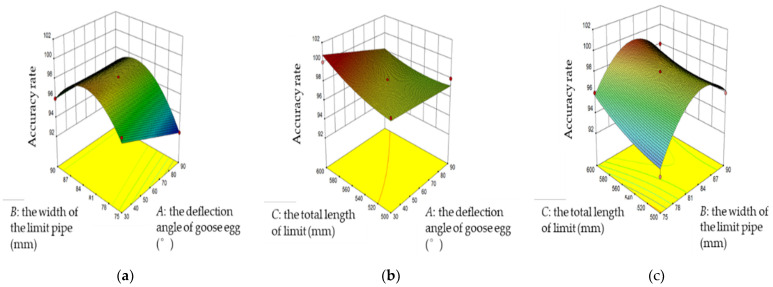
The 3D response surface diagram of interaction effects of various factors: (**a**) relationship among accuracy, width of the limit pipe, and deflection angle of goose egg; (**b**) relationship among accuracy, total length of the limit device, and deflection angle of goose egg; (**c**) relationship among accuracy, total length of the limit device, and width of the limit pipe.

The egg body limit transmission device was the premise to realize the complete coding of goose eggs. A smaller deviation between the long axis direction of goose eggs and the conveying direction of induction conveyor belt results in a higher coding success rate. Therefore, in order to determine the optimal scheme of the limit scheme, 50 goose eggs were randomly selected for attitude adjustment through an orthogonal rotation test. The test results are shown in Table 1. The variance analysis of the success rate model of attitude adjustment was carried out. The 3D response surface diagram of the interaction effect of various factors is shown in Figure 7. The success rate of attitude adjustment decreased with the increase in deflection angle. The goose egg posture adjustment was correct and increased with the increase in limit width. After reaching the extreme point, the goose egg posture adjustment was successful and decreased with the increase in limit width; The successful attitude adjustment increased with the increase in the total length of the limit device.

In this study, a maximum deflection angle of goose egg of 90° was selected as the condition, and the regression model of goose egg attitude adjustment success rate was solved. The following optimal parameters were obtained: limit width of 82.5 mm, total length of the limit device of 600 mm, and attitude adjustment success rate of 98.25%.

### 3.2. Comparison of Target Detection Algorithms

**Figure 8:**
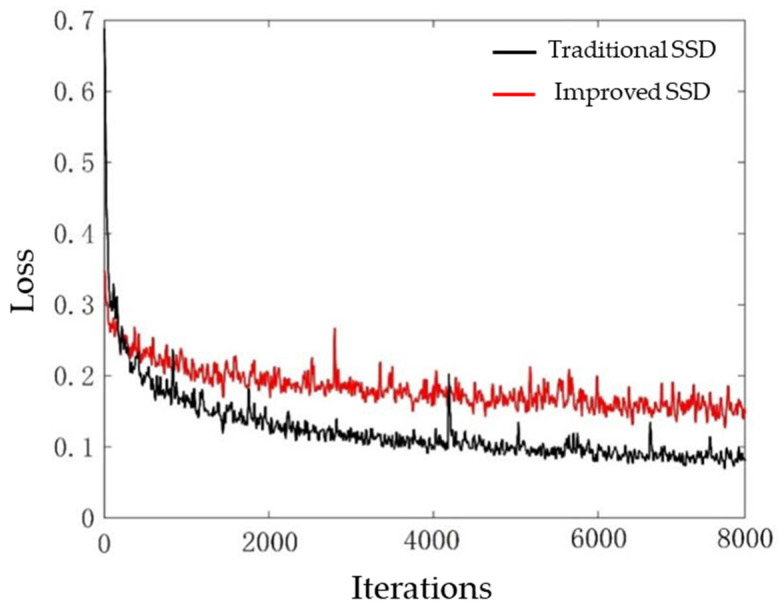
Comparison of loss values of two algorithms.

The target detection module in this study used HIKVISION DS-IPC-T12H2-I/POE cameras, which were installed on the top of the delivery rooms for shooting. The videos taken in the delivery rooms were sampled and divided into frames, and the divided images were used as the datasets required for the experiment. A total of 5600 images were divided into a training set (4800 images), verification set (300 images), and test set (500 images). Labimg was used to label the breeding geese and goose eggs in the images, and the labeling information was saved in “.XML” format. The computer configuration was as follows: Intel i7-8750 CPU, Ubuntu 16.04 operating system, and NVIDIA GTX1060ti. The experimental framework adopted Pytoch deep learning frame. The datasets were imported into the traditional SSD model and improved SSD for training. In the training process, the weight parameters were updated by random gradient descent. The momentum term was set to 0.9, the initial learning rate was set to 0.001, the decay parameter was set to 0.0005, and the number of iterations was 8000. Figure 8 shows the loss rate of the two algorithms. It can be seen from Figure 8 that the loss value of the model under the two algorithms dropped rapidly after several iterations, and then the convergence of the loss value slowed down and gradually tended to be flat. Although the overall oscillation amplitude of the traditional SSD algorithm and the improved SSD algorithm was small, the convergence speed of the improved SSD target monitoring algorithm was faster, and the loss value was lower than that of the traditional SSD target detection algorithm.

**Figure 9:**
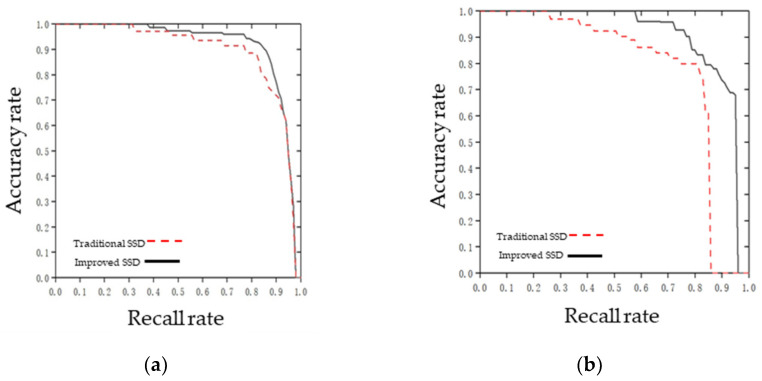
Comparison of accuracy–recall curves under two algorithms of breeding geese and goose egg: (**a**) breeding geese; (**b**) goose eggs.

At the same time, in order to verify the detection effect of the two models, after the model training, the test dataset was imported into the traditional SSD model and the improved SSD model for test and comparison. The IOU value was set to 0.5, and the accuracy–recall curves of breeding geese and goose eggs is shown in Figure 9. It can be seen from Figure 9a that, with the improvement of recall rate, the detection accuracy of breeding geese was slightly higher than that of the traditional SSD target detection algorithm. As can be seen from Figure 9b, the goose egg detection accuracy was higher than the traditional SSD target detection algorithm as a whole. Compared with the traditional SSD, the improved SSD in this study had a higher recognition rate for small target objects such as goose eggs, which was more suitable for the needs of target detection in this study.

**Figure 10:**
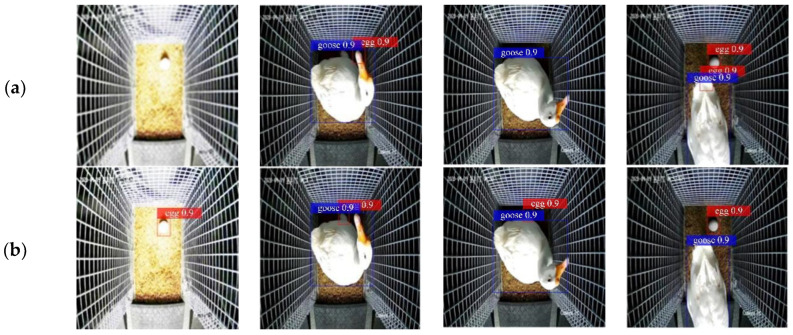
Some effects of the two algorithms on target recognition: (**a**) traditional SSD; (**b**) improved SSD.

Some effects of the two algorithms on target recognition are shown in Figure 10. In Figure 10, it can be seen that, compared with the traditional SSD, the improved SSD proposed in this paper could successfully recognize the targets in the delivery rooms, especially for small target objects such as goose eggs. We digitized the test results, and the accuracy results are shown in Table 2. It can be seen from Table 2 that there was little difference in the target detection accuracy between the SSD target detection algorithm and the *MAP* value of the improved SSD. This was because breeding geese accounted for a large proportion in the video and were easier to detect. The goose egg target detection accuracy in the improved SSD target detection algorithm was greater than that in the SSD target detection algorithm, 8.65% higher, and the detection speed was also improved by 62.6 ms. Therefore, the improved SSD target detection algorithm proposed in this study was better than the traditional SSD model in the real-time detection of geese and goose eggs in the delivery rooms, meeting the detection needs of the delivery rooms.

### 3.3. Device Coding Experiment

In order to adapt the breeding goose to the goose breeding egg identification and coding system, we placed the prototype of the identification and coding system in the breeding goose breeding base for 14 days, during which only the target detection module was opened to collect the images of breeding geese and goose eggs. Then, we turned on the system for the goose egg coding experiment. The test results are shown in Table 3. A group of six breeding geese and 127 breeding eggs were analyzed. The success rate of egg laying and coding of breeding geese 1, 3, and 5 was 100%, realizing the one-to-one correspondence between the identity of breeding eggs and breeding geese. The goose eggs of other geese failed to code. Overall, the average success rate of goose egg coding was 97.8%. The reason for the failure of inkjet marking was that the size of individual goose eggs was relatively small, resulting in a poor inkjet effect. In addition to the unusual size of goose eggs, the success rate of inkjet coding of the system is high, which met the requirements of goose egg coding identification and individual traceability function.

**Figure 11:**
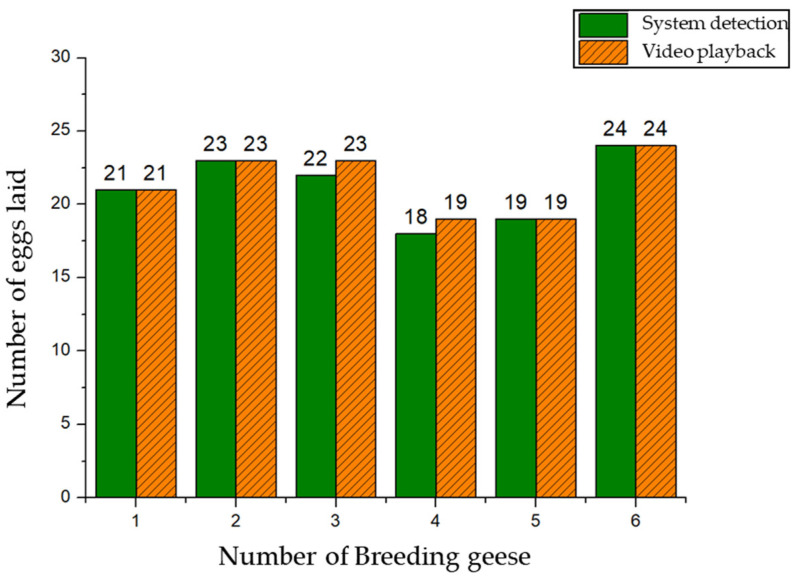
Comparison of egg production in different recording methods.

At the same time, in order to verify whether the actual egg-laying information of breeding geese corresponded to the information on goose eggs, we performed video playback calibration on the egg-laying records of breeding geese, and the results are shown in Figure 11. It can be seen from Figure 11 that the egg production observed by video playback of breeding geese 1, 2, 5, and 6 was consistent with the egg production data monitored by the system. The egg production data of breeding geese 3 and 4 were recorded incorrectly. The reason was that breeding geese No. 4 immediately entered the production room to lay eggs after finishing the egg production operation. After breeding goose 3 left, the system detected two goose eggs and triggered an alarm. The integrated production room device stopped operation, and the egg production data were not recorded on the goose eggs. As a result, some goose eggs did not form a corresponding relationship with breeding geese.

## 4. Conclusions

In this study, a goose breeding egg identification and coding system for selection of high-quality breeding geese was developed. The system could identify and code the breeding eggs in time and help the breeders get the correspondence between breeding goose individual information and egg-laying information through the QR codes on the eggs. The evaluative experiment results showed that the pass rate of egg body coding reached 98.25%, the proposed improved SSD algorithm was 8.65% more accurate and 62.6 ms faster than the traditional SSD, and the accuracy rate corresponding to the individual information of the breeding goose and the surface information of the goose egg was 97.8%. The system we developed still has some defects; when the egg-laying interval between two geese is too small, the system cannot judge the breeding goose information corresponding to the two kinds of eggs in the delivery room, resulting in a recording error.

In a future study, we will focus on optimizing of the device structure to automatically adapt to different goose egg sizes and improving the adaptive ability of the system to possible abnormal conditions.

## Figures and Tables

**Table 1:** Attitude adjustment results of limit devices with different sizes.

Experience Group	A (°)	B (mm)	C (mm)	η **(%)**
1	60	75	600	96
2	30	90	550	96
3	60	75	500	92
4	60	82.5	550	98
5	60	82.5	550	98
6	60	82.5	550	98
7	90	75	550	92
8	60	90	600	98
9	90	82.5	500	98
10	30	75	550	96
11	30	82.5	600	100
12	60	90	500	96
13	60	82.5	550	98
14	30	82.5	500	98
15	60	82.5	550	98
16	90	90	550	96
17	90	82.5	600	98

Note: A, the deflection angle of the goose egg; B, the width of the limit pipe; C, the total limit length.

**Table 2:** Detection results of breeding geese and goose eggs.

Models	*AP* (%)		*MAP* (%)	Speed (ms)
	Breeding Geese	Goose Eggs		
Traditional SSD	87.41%	75.81%	81.61%	10.2
Improved SSD	92.58%	87.94%	90.26%	72.8

**Table 3:** The results of goose eggs coding.

ID	Number of Collected Goose Eggs	Number of Coded Goose Eggs	Number of Qualified Eggs	Coding Success Rate
1	21	21	21	100%
2	23	23	22	95.7%
3	22	22	21	95.5%
4	18	18	18	100%
5	19	19	19	100%
6	24	24	23	95.8%
Average	21.1	21.1	20.7	97.8%

## Data Availability

Not applicable.
